# Phenolic Profiling of the South American “Baylahuen” Tea (*Haplopappus* spp., Asteraceae) by HPLC-DAD-ESI-MS

**DOI:** 10.3390/molecules20010913

**Published:** 2015-01-08

**Authors:** Guillermo Schmeda-Hirschmann, Cristina Quispe, Benita González

**Affiliations:** 1 Laboratorio de Química de Productos Naturales, Instituto de Química de Recursos Naturales, Universidad de Talca, Casilla 747, Talca, Chile; E-Mail: equispe@utalca.cl; 2 Facultad de Ciencias Agrarias, Universidad de Talca, Casilla 747, Talca, Chile; E-Mail: bgonzalez@utalca.cl

**Keywords:** baylahuen, *Haplopappus* spp., South American herbal tea, phenolics, HPLC-DAD-MS, chemotaxonomy

## Abstract

The aerial parts of several *Haplopappus* species (Asteraceae), known under the common name “baylahuen”, are used as herbal teas in Chile and Argentina. In Chile, “baylahuen” comprises *H. multifolius*, *H. taeda*, *H. baylahuen* and *H. rigidus*. Little is known about the chemical identity of the infusion constituents in spite of widespread consumption. The aim of the present work was the characterization of phenolics occurring in the infusions and methanol extracts of “baylahuen” by HPLC-DAD-ESI-MS. A simple HPLC-DAD-ESI-MS method was developed for the fast identification and differentiation of *Haplopappus* spp. used as a tea source, based on the phenolics from the tea and methanol extracts. Some 27 phenolics were tentatively identified in the infusions and methanol extract, including 10 caffeoyl quinic and feruloyl quinic acid derivatives and 17 flavonoids. The HPLC patterns of the *Haplopappus* tea and methanol extract allow a clear differentiation at the species level. The occurrence of hydroxycinnamic acid derivatives and flavonoids can explain the reputed nutraceutical and health beneficial properties of this herbal tea.

## 1. Introduction

**Figure 1:**
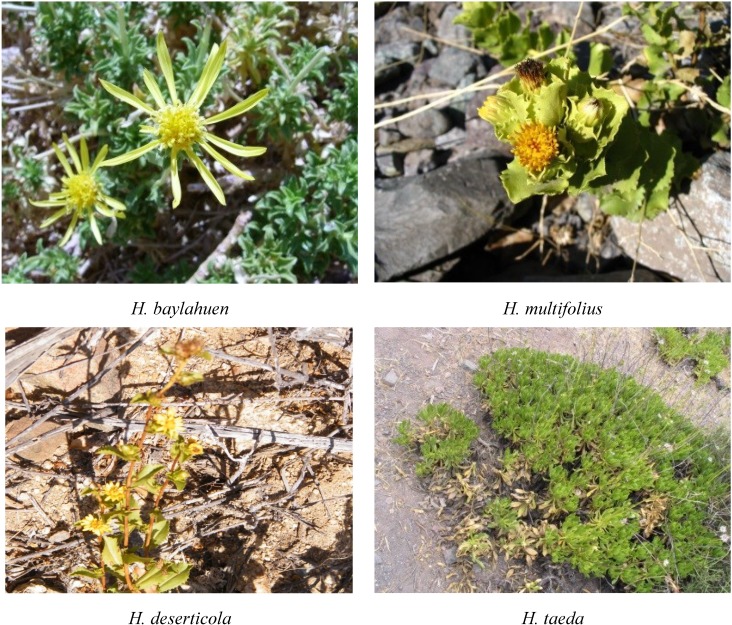
Chilean *Haplopappus* spp. belonging to the “baylahuen” complex growing in their natural habitat.

Herbal teas are considered as a healthy alternative, because of the beneficial effects of nutraceutical antioxidants present in them, as well as other properties associated with traditional lore. The aerial parts of several Asteraceae, known under the common name “baylahuen”, are used in Chile and Argentina as a functional tea for its liver stimulating properties and pleasant taste. The “baylahuen” complex in Chile comprises *H. baylahuen*, *H. multifolius*, *H. rigidus* and *H. taeda* (Figure 1). The related species, *H. deserticola*, can be collected by mistake.

Polyphenols are some of principal compounds related to the benefits of fruits, vegetables, wine and plant tea consumed in the diet, due to their antioxidant properties. Plant phenolics are also useful as chemotaxonomic markers [1,2]. The polyphenols are recognized for their nutritional value, since they may help reduce the risk of chronic disease and have a positive effect on health [2,3]. All of these properties are strongly dependent on the polyphenol chemical structure [4]. Numerous analytical procedures have been proposed for the analysis of phenolic compounds employing various extraction, separation and quantification techniques. Liquid chromatography with diode array detection with tandem mass spectrometry (HPLC-DAD-MS) is a suitable technique for the fast identification of constituents in complex mixtures and to obtain tentative structures of phenolic compounds in plant extracts [5]. It has been successfully applied to metabolome analysis and is a powerful tool for the fast characterization of phenolic compounds in edible fruits [6,7,8], crop plants and herbal teas [9,10,11,12], as well as marine microalgae [13].

The antioxidant activity and thin layer chromatography (TLC) characterization of five “baylahuen” species, as well as the biomass and resin content in different natural populations was reported [14,15]. However, the identity of the compounds was not reported in the TLC comparison. The chemistry of *Haplopappus* species has been investigated mainly for the exudate composition and lipophilic compounds [16,17,18,19]. Little is known about the chemical identity of the constituents occurring in the “baylahuen” tea or its polar extracts. Both glycosides and free phenolics belonging to the flavonoids, coumarins, phenolic acids and tannins occurs in variable amounts in herbal teas, and several of them present relevant biological activities. The aim of the present work was the characterization of the phenolics occurring in the methanol extracts and infusions (tea) of *H. baylahuen*, *H. deserticola*, *H. multifolius*, *H. rigidus* and *H. taeda* by HPLC-DAD-ESI-MS. The phenolics composition was compared with total phenolic and total flavonoid content and with the antioxidant activity of the plant extracts [14]. This information can be a useful reference for nutraceutical polyphenols in “baylahuen” tea, for quality control and for chemotaxonomic purposes.

## 2. Results and Discussion

### 2.1. TP, TF and DPPH^−^ Analysis

**Figure 2:**
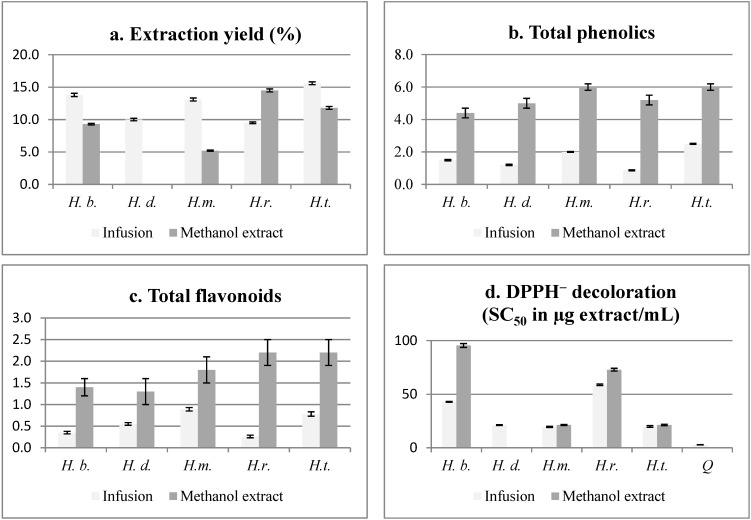
Comparison of the infusion and methanol extract from the aerial parts of “baylahuen” (*Haplopappus* spp.) in the following parameters: (**a**) percent *w*/*w* extraction yield; (**b**) total phenolics (TP); (**c**) total flavonoids (TF); and (**d**) scavenging of the free radical, DPPH (SC_50_ (scavenging concentration of 50%)). H.b., *H. baylahuen*; H.d., *H. deserticola*; H.m., *H. multifolius*; H.r., *H. rigidus*; H.t., *H. taeda.* All measurements are expressed as the mean ± S.D. (*n* = 3). The yield of the extractions is expressed as g of extract per 100 g of dry plant weight. TP and TF are calculated on the basis of the dry plant. TP is expressed as g of gallic acid equivalents/100 g of dry plant. Antiradical DPPH^−^ decoloration activity is expressed as SC_50_ in µg of dry extract/mL. The yield of the extractions is expressed as g of lyophilized infusion per 100 g of dry plant. n.d., not done.

Infusions (tea) and methanol extracts of the five *Haplopappus* species, commercialized as the herbal tea “baylahuen”, were compared for total phenolics, total flavonoids, scavenging of the DPPH radical and extraction yield (Figure 2). The highest free radical scavenging effect in the infusions was found for *H. deserticola*, *H. multifolius* and *H. taeda* with SC_50_ (scavenging concentration of 50%) of 21.2, 19.5 and 20.0 µg/mL, respectively. The tea of the three mentioned species also showed higher total flavonoid content, while *H. taeda*, *H. multifolius* and *H. baylahuen* presented higher TP.

The yield of extraction for the infusion was between 9.5%–15.6% and for the methanol extract 5.2%–14.5%, respectively. The extraction yield is relatively high compared with the infusion of other Chilean medicinal plants [20]. For the methanol extracts, *H. multifolius* and *H*. *taeda* presented a good free radical scavenging effect with SC_50_ of 21.3 µg/mL and with higher total flavonoids (TF) and total phenolics (TP) content than the corresponding infusions (Figure 2). While the TP/TF ratio for the infusions shows a general trend with the DPPH bleaching activity, there is no clear relation with the methanol extracts.

### 2.2. HPLC-DAD-MS Analysis

**Figure 3:**
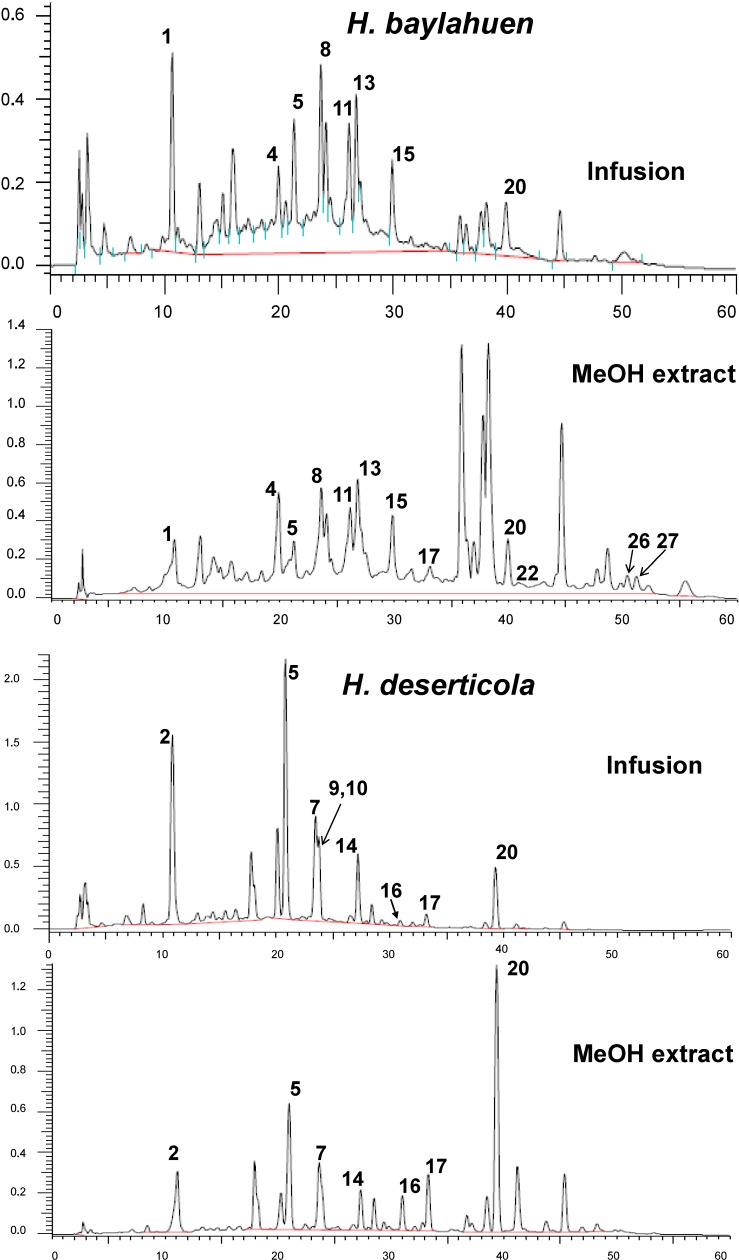

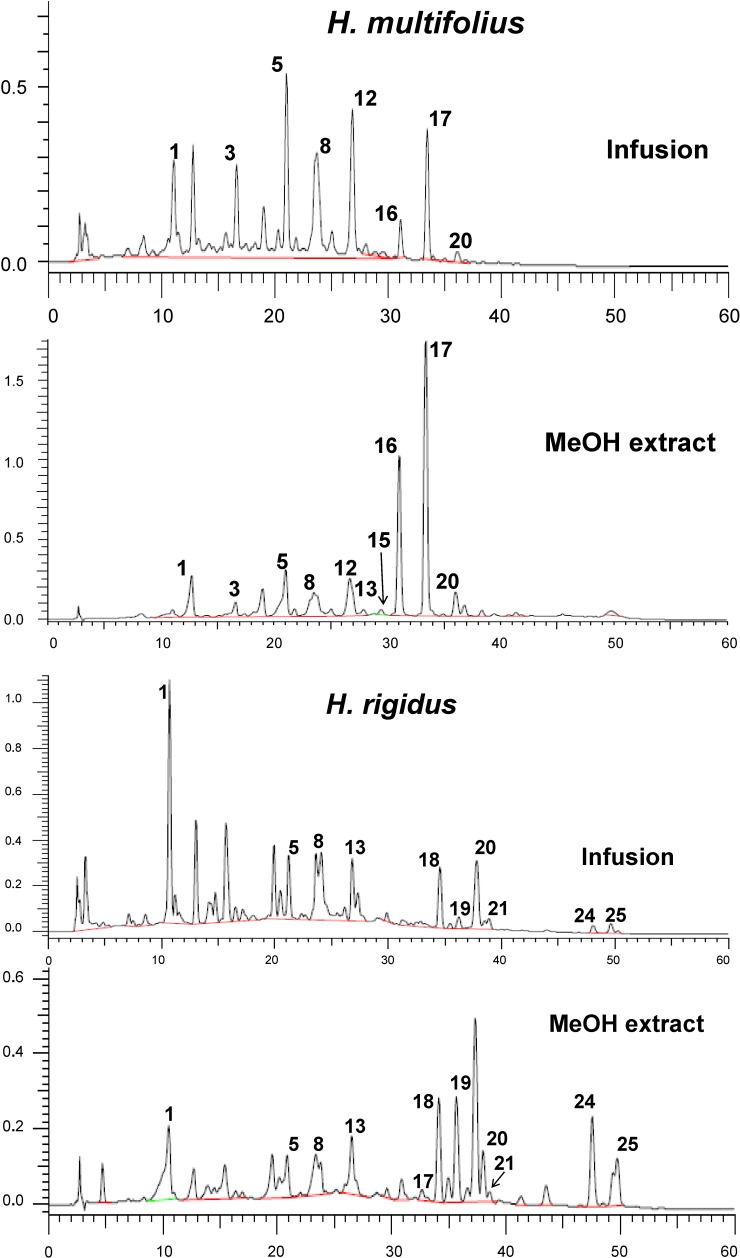

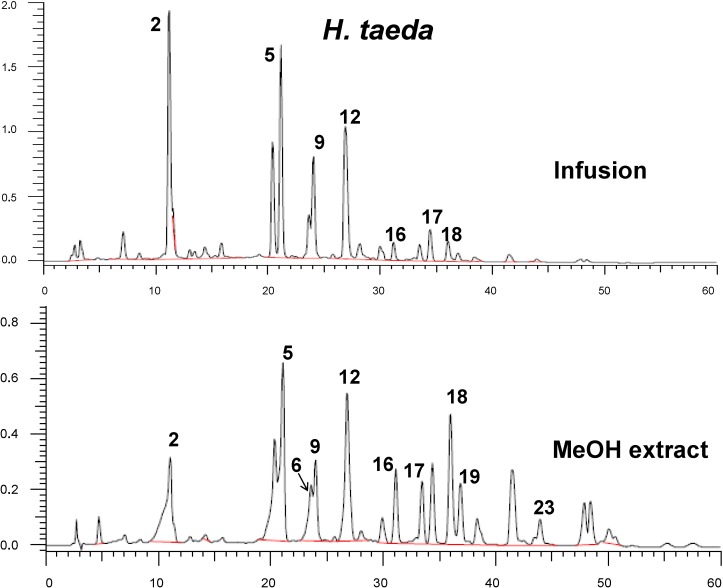
Comparative HPLC-DAD chromatograms at 250 nm of “baylahuen” (*Haplopappus* spp.) infusions (tea) and methanol extracts. For the tentative identification of the constituents, please see Table 1 and Table 2.

In this study, using the negative ESI mass detection mode, phenolics were tentatively identified in teas and methanol extracts from *Haplopappus* spp. Comparative HPLC-DAD chromatograms at 250 nm of “baylahuen” (*Haplopappus* species) methanol extracts and teas (infusions) are presented in Figure 3. The retention time (Rt), UV spectral maxima, MS fragmentation and tentative identification of the compounds are summarized in Table 1. The distribution of the phenolic constituents in the *Haplopappus* species investigated are shown in Table 2.

**Table 1:** Identification of phenolic compounds in *Haplopappus* spp. tea and methanol extract by HPLC-DAD-ESI-MS data.

Peak	R_t_ (min)	UV λ_max_ (nm)	MW	[M−H]‾ and Ions	Tentative Identification
1	10.9	323, 300 sh, 238	368	367, 193, 134	Feruloylquinic acid
2	11.3	325, 300 sh, 240	354	353, 191, 173	Caffeoylquinic acid (Chlorogenic acid)
3	16.6	-	530	529, 501, 367, 193	Feruloyl caffeoylquinic acid isomer
4	19.6	321, 300 sh, 236	706	705, 543, 367, 349, 193, 173	Caffeoyl diferuloylquinic acid
5	21.1	327, 300 sh, 242	516	515, 353, 173; MS^2^ 353, 203, 191	3,5-Dicaffeoylquinic acid
6	23.5	349, 320 sh, 245	580	579, 447, 285	Kaempferol 3-*O*-hexoside pentoside
7	23.7	352, 300 sh, 260 sh, 255	610	609, 477, 315	Quercetin methyl ether pentoside hexoside
8	23.8	323, 300 sh, 242	530	529, 367, 173	Feruloyl caffeoylquinic acid isomer
9	24.0	327, 300 sh, 243	516	515, 353, 173; MS^2^ 353, 299, 203	3,4-Dicaffeoylquinic acid
10	24.0	-	478	477, 315, 301, 153	Quercetin methyl ether hexoside
11	26.2	323, 300 sh, 239	544	543, 349, 193, 173	Diferuloylquinic acid isomer
12	26.7	344, 300 sh, 265	448	447, 285	Kaempferol-3-*O*-hexoside
13	26.8	325, 300 sh, 242	530	529, 367, 173	Feruloyl caffeoylquinic acid isomer
14	27.2	355, 300 sh, 260 sh, 253	492	491, 315	Quercetin methyl ether glucuronate
15	29.8	325, 300 sh, 239	544	543, 367, 349, 193, 173	Diferuloylquinic acid isomer
16	31.1	370, 300 sh, 260 sh, 253	302	301, 179, 151, 107	Quercetin
17	33.4	358, 300, 260 sh, 255	316	315, 300, 271, 255, 179	Quercetin 3-*O*-methyl ether
18	36.0	290, 235	302	301, 273, 165	Trihydroxy methoxyflavanone
19	36.9	364, 320 sh, 265	286	285, 268, 257	Kaempferol
20	38.5	346, 296, 253, 231	330	329, 314, 176; 176, 148, 104	Quercetin dimethyl ether
21	39.4	-	360	359, 344, 329, 317, 299	Quercetin tetramethyl ether
22	39.9	349, 330 sh, 300 sh, 267	300	299, 284, 271, 166	Kaempferol methyl ether
23	43.9	287, 231	316	315, 300, 193, 165	Dihydroxy dimethoxyflavanone
24	47.6	364, 320sh, 265	300	299, 284, 271, 165	Kaempferol 3-*O*-methyl ether
25	49.5	349, 369	314	313, 298, 283	Kaempferol dimethyl ether
26	50.4	-	378	377, 317, 275, 257	Myricetin monoacetate
27	51.2	-	320	319, 301, 273	Dihydromyricetin

-, not measured due to the low amount of the compound.

**Table 2:** Distribution of the phenolic compounds in “baylahuen” tea and methanol extract by HPLC-DAD-ESI-MS data.

Compound	Tentative Identification	*H. baylahuen*	*H. deserticola*	*H. multifolius*	*H. rigidus*	*H. taeda*
1	Feruloylquinic acid isomer	x		x	x	
2	Caffeoylquinic acid (chlorogenic acid)		x			x
3	Feruloyl caffeoylquinic acid isomer			x		
4	Caffeoyl diferuloylquinic acid	x				
5	3,5-Dicaffeoylquinic acid	x	x	x	x	x
6	Kaempferol 3-*O*-hexoside pentoside					x
7	Quercetin methyl ether pentoside hexoside		x			
8	Feruloyl caffeoylquinic acid isomer	x		x	x	
9	3,4-Dicaffeoylquinic acid		x			x
10	Quercetin methyl ether hexoside		x			
11	Diferuloylquinic acid isomer	x				
12	Kaempferol-3-*O*-hexoside			x		x
13	Feruloyl caffeoylquinic acid isomer	x		x	x	
14	Quercetin methyl ether glucuronate		x			
15	Diferuloylquinic acid isomer	x		x		
16	Quercetin		x	x		x
17	Quercetin 3-*O*-methyl ether	x	x	x	x	x
18	Trihydroxy methoxyflavanone				x	
19	Kaempferol				x	x
20	Quercetin dimethyl ether	x	x	x	x	
21	Quercetin tetramethyl ether				x	
22	Kaempferol methyl ether	x				
23	Dihydroxy dimethoxyflavanone					x
24	Kaempferol 3-*O*-methyl ether				x	
25	Kaempferol dimethyl ether				x	
26	Myricetin monoacetate	x				
27	Dihydromyricetin	x				

### 2.3. Caffeoylquinic Acid Derivatives

Several hydroxycinnamates, including feruloyl and caffeoyl quinic acid derivatives, were found in all of the samples investigated. The UV spectral data, fragmentation patterns and chromatographic behavior were compared with the literature for the tentative assignment [21,22,23,24,25]. Compounds **1** and **2** are in agreement with feruloyl and caffeoylquinic acid isomers, respectively. Compound **2** was identified as chlorogenic acid by comparison with a standard sample. The mass spectra of Compounds **3**, **8** and **13** show consecutive loss of 162 amu (atomic mass units) followed by 194 amu, in agreement with a caffeoyl and a feruloyl moiety attached to the quinic acid, which were identified as feruloyl caffeoylquinic acid isomers. The UV maxima for Peaks **5** and **9**, as well as the MS spectra are in agreement with dicaffeoylquinic acid isomers [21,23,25]. A comparison of the MS^2^ spectra of Compounds **5** and **9** shows the characteristic fragments of 3,5 and 3,4-dicaffeoylquinic acid, respectively [25]. Compounds **11** and **15**, with a [M−H]^−^ ion at *m*/*z* 543 showed a loss of 176 amu (ferulate), leading to the ion at 367, which, in turn, loses water and the second ferulate unit. The proposed fragmentation is in agreement with that reported by [21,25,26] for similar compounds. The UV spectrum of Compound **4** shows a maxima at λ 321 nm, in the same range as the dicaffeoyl-, diferuloyl- and feruloyl caffeoylquinic acid isomers occurring in the extracts. The mass spectrum with a [M−H]^−^ ion at *m*/*z* 705 showed the loss of 162 amu leading to a [M−H]^−^ ion at *m*/*z* 543, which displays the same fragmentation pattern as the diferuloylquinic acid isomers, **11** and **15**. Compound **4** was tentatively assigned to a caffeoyl diferuloylquinic acid derivative. However, isolation and spectroscopic studies are needed for a full characterization.

### 2.4. Flavonoids

Compounds **6**, **7**, **10**, **12**, **14** and **16**–**27** were identified as flavonoids based on the shape of the UV spectra [27] and MS data. Compounds **7**, **10**, **14**, **16**, **17** and **20** presented UV maxima in agreement with a flavonol or a 3-*O*-substituted flavonol and were identified as quercetin or methyl quercetin derivatives. Compound **7** with a [M−H]^−^ ion at *m*/*z* 609 fragmented to a methyl quercetin aglycone at *m*/*z* 315 after neutral loss of a pentose (132 amu) and a hexose (162 amu). The related Compound **10** showed the loss of a hexose (162 amu) and **14** the loss of glucuronic acid (176 amu), leading to the same aglycone at *m*/*z* 315. Compound **16** was identified as quercetin, **17** as quercetin-3-*O*-methyl ether and **20** as quercetin dimethyl ether on the basis of the UV spectra and MS fragmentation [28]. Compound **21** was tentatively assigned to quercetin tetramethyl ether. The UV spectrum of Compounds **19** and **24** showed a UV maximum of 364 nm, compatible with a flavonol with a free 3-hydroxy function, while Compounds **6**, **12**, **22** and **25**, with UV maxima of 344 to 349 nm, are in agreement with a 3-*O*-substituted flavonol. Compound **6** showed neutral loss of 132 and 162 amu (pentose and hexose), leading to kaempferol MS ions at *m*/*z* 285 and *m*/*z* 255, while Compound **12** with a [M−H]^−^ ion at *m*/*z* 447 fragmented to a kaempferol aglycone at *m*/*z* 285 [28]. Compounds **6** and **12** were tentatively identified as kaempferol 3-*O*-hexoside pentoside and kaempferol 3-*O*-hexoside, respectively. Compound **19** was identified as the aglycone kaempferol, while Compounds **22** and **24**, with a [M−H]^−^ ion at *m*/*z* 299 and differing in the UV maxima, were assigned as kaempferol methyl ether and kaempferol 3-*O*-methyl ether, respectively. Peak **25**, with a [M−H]^−^ ion at *m*/*z* 313 showed two consecutive losses of 15 amu, in agreement with a dimethyl ether of kaempferol. The minor constituents, **26** and **27**, were tentatively identified as myricetin monoacetate and dihydromyricetin, respectively. The UV spectra of Compounds **18** and **23** showed maxima of 287–290 nm, compatible with a flavanone. Both compounds differ in the molecular weight (302 and 316 amu, respectively) and presented similar fragmentation patterns, leading to the *m*/*z* ion at 165 amu. The compounds were tentatively assigned as trihydroxy methoxyflavanone and dihydroxy dimethoxyflavanone, respectively [29].

### 2.5. Distribution of the Phenolic Compounds

Both the “baylahuen” tea, as well as the methanol extracts present the same constituents, but differ in their relative proportion. From the 27 compounds tentatively identified in the tea and extract, ten of them were hydroxycinnamic acid derivatives and seventeen were flavonoids. Dicaffeoyl quinic acid and quercetin methyl ether occur in all samples. Compounds **1**–**5**, **8**, **9**, **11**, **13** and **15** were identified as caffeoyl- or feruloylquinic acid derivatives. Seven of them (Compounds **1**, **4**, **5**, **8**, **11**, **13** and **15**) occurred in *H. baylahuen*, six in *H. multifolius* (**1**, **3**, **5**, **8**, **13** and **15**), four in *H. rigidus* (**1**, **5**, **8** and **13**) and three in *H. taeda* and *H. deserticola* (**2**, **5** and **9**). Several quercetin derivatives were tentatively identified in *H. deserticola* (Peaks **7**, **10**, **14**, **16**, **17** and **20**), three in *H. multifolius* (**16**, **17** and **20**) and *H. rigidus* (**17**, **20** and **21**), two in *H. baylahuen* (peaks **17** and **20**) and *H. taeda* (**16** and **17**). The only species showing quercetin glycosides was *H. deserticola*, while quercetin aglycones occurred in all of the species investigated. Kaempferol derivatives were identified in *H. rigidus* (Peaks **19**, **24** and **25**), *H. taeda* (**6**, **12** and **19**) and *H. multifolius* (Peak **12**). The flavonoids tentatively identified in the samples were mainly flavonol aglycones and glycosides. The aglycones were mainly kaempferol, quercetin and its mono- and di-methyl ethers.

Hydroxycinnamic acid derivatives are the main constituents of the infusions, and flavonoid aglycones are more abundant in the methanol extracts. Closer HPLC patterns for *H. deserticola* and *H. taeda* indicate high similarity in phenolics with caffeoyl- and feruloylquinic acid derivatives as the main constituents. In a study on the antioxidant effect of Chilean herbal teas [30], *H. baylahuen* infusion was one of the most effective regarding Trolox-equivalent antioxidant capacity (TEAC) and HClO^−^ quenching activities. According to this reference, about 150 mL of baylahuen tea were equivalent to about 200 mg of Trolox. Our findings further support the results [14,30] and present for the first time the tentative characterization of the main antioxidant phenolics in the tea and polar extract of the plant sources of “baylahuen”.

## 3. Experimental Section

### 3.1. Plant Material

The plants used for the present study were collected in northern Chile, as described by [15]. *Haplopappus baylahuen* Remy, *H. rigidus* Phil. and *H. deserticola* Phil. were collected in the Region de Atacama, *H. multifolius* Philippi ex Reiche in the Region de Valparaiso and *H. taeda* Reiche in the Region del Libertador Bernardo O`Higgins. Voucher herbarium specimens have been deposited at the Herbario de la Universidad de Talca. *H. deserticola* was collected in February, 2010, and all other herbs were collected between January and March, 2005. The plant sources of the tea, *H. multifolius*, *H. baylahuen*, *H. taeda* and *H. deserticola*, are shown in Figure 1.

### 3.2. Chemicals

Folin–Ciocalteu phenol reagent, 1,1-diphenyl-2-picrylhydrazyl radical (DPPH), sodium nitrite, sodium hydroxide, gallic acid, quercetin, rutin and chlorogenic acid (3-*O*-caffeoylquinic acid) were purchased from Sigma-Aldrich Chemical Co. (St. Louis, MO, USA). HPLC-grade acetonitrile, methanol and HPLC-grade water were obtained from J.T. Baker (Phillipsburg, NJ, USA). Analytical grade ethyl acetate, chloroform, acetic acid, formic acid, sodium carbonate, 2-aminoethyldiphenyl borate were from Merck (Darmstadt, Germany). The purity of the chemicals used was as follows: sodium hydroxide reagent grade, ≥98%, sodium nitrite American Chemical Society (ACS) reagent ≥ 97.0%, gallic acid (purity ≥ 99%), quercetin (purity ≥ 97%), chlorogenic acid (purity ≥ 95%), Folin–Ciocalteu phenol reagent (2 M, with respect to acid, Sigma) and 1,1-diphenyl-2-picrylhydrazyl radical (DPPH, 95% purity) (Sigma-Aldrich).

### 3.3. Preparation of Extracts

The plant samples were dried at room temperature (about 30 °C) for two weeks, powdered and extracted as follows. The tea was prepared adding 50 mL of boiling water to 1.5 g of the powdered herb, and the infusion was allowed to cool throughout the extraction process to mimic tea brewing. After filtering, the infusion was frozen and lyophilized using a Labconco Freeze Dry System. For the methanol extracts, the powdered samples (1.7 g) were extracted with methanol (10 mL) at room temperature under sonication (5 min), filtered and taken to dryness to afford the methanol extracts. Dry extracts were kept in a freezer for further analysis. The extraction yield was calculated as g of extract per 100 g of dry plant weight.

### 3.4. Total Phenolics and Total Flavonoids Content

The total phenolic content (TP) was determined by the Folin–Ciocalteu method [8,10,11]. All samples and gallic acid were dissolved in 50% (*v*/*v*) aqueous methanol. Samples (50 µL) were placed into test tubes, and 250 μL of Folin–Ciocalteu reagent were added. The mixture was left to stand for 5 min, and 750 µL of 20% sodium carbonate solution and 5 mL distilled water were added. After 30 min of incubation at room temperature (20 °C), the absorbance of the solution was measured at 765 nm. The calibration curve was performed with gallic acid (concentrations ranging from 31.3 to 500.0 µg/mL), and the results are expressed as mg of gallic acid equivalents per 100 g of dry plant material. The TF content in the samples was determined according to [31] using quercetin as the reference for the calibration curve. The absorbance of the reaction mixture was measured at 415 nm, and the results are expressed as g of quercetin equivalents per 100 g of dry weight. Data are reported as the mean ± SD for at least three replications.

### 3.5. Scavenging of DPPH Radicals

The scavenging of DPPH radicals was assayed as previously reported [8,10,11]. All extracts were dissolved in 50% (*v*/*v*) aqueous methanol to prepare stock solutions of 1 mg/mL. The stock solutions were serially diluted with methanol, mixed with an equal volume of DPPH solution (60 µM) and shaken vigorously. The mixture was incubated at room temperature for 30 min before the absorbance at 517 nm was read. Solutions of quercetin were used as a positive control. The scavenging activity was determined by comparing the absorbance of the samples with that of the blank (100%) that contained only DPPH and solvent. Antiradical DPPH^−^ bleaching activity is expressed as SC_50_ (scavenging concentration of 50%) of the infusions and extracts in µg/mL, which denoted the concentration of sample required to scavenge 50% of DPPH radicals. SC values lower than 50 µg/mL are considered high, while values between 50 and 100 µg/mL are considered moderate.

### 3.6. HPLC-DAD Analysis

The HPLC system used for DAD analysis of the samples consisted of Merck-Hitachi (LaChrom, Tokyo, Japan) equipment consisting of an L-7100 pump, an L-7455 UV diode array detector and a D-7000 chromatointegrator. A 250 mm × 4.60 mm i.d., 5-μm Kromasil 100-5C18 column (Eka Chemicals, Brewster, NY, USA) maintained at 25 °C was used. Approximately 5 mg of each extract, obtained as explained above, were dissolved in 1 mL methanol/water (1:1 *v*/*v*), filtered through a 0.45-µm PTFE filter (Alltech Associates Inc., Deerfield, IL, USA) and submitted to HPLC-DAD and HPLC-MS analysis. The compounds were monitored at 250 nm, and UV spectra from 200 to 600 nm were recorded for peak characterization. The HPLC analysis was performed using a linear gradient solvent system consisting of 1% formic acid (A) and methanol (B) as follows: 75% to 40% A over 25 min; followed by 40% to 25% A from 25 to 45 min; 25% to 40% A from 45 to 60 min. The flow rate was 1 mL/min. For the analysis, 5 mg of the lyophilized extract were dissolved in 1 mL methanol/water (1:1 *v*/*v*). The volume injected was 20 µL. An Inertsil ODS-3 column (250–4.6 mm; particle size: 5 µm) (GL Sciences Inc., Tokyo, Japan) was used for the comparison of infusions and methanol extracts of the plants.

### 3.7. ESI-MS/MS Analysis

Mass spectra were recorded using an Agilent 1100 (USA) liquid chromatography system connected through a split to an Esquire 4000 Ion Trap LC/MS system (Bruker Daltoniks, Germany) or a Merck-Hitachi 6200 Intelligent Pump and L 4000 UV detector coupled to a EBE trisector VG Autospec Micromass spectrometer (Micromass-Water Autospec, Manchester, UK) operating at 70 eV. Full scan mass spectra were measured between *m*/*z* 150 and 2000 u in negative ion mode. For the ion trap LC/MS system, nitrogen was used as the nebulizer gas at 27.5 psi, 350 °C and at a flow rate of 8 L/min. The mass spectrometric conditions for negative ion mode were: electrospray needle, 4000 V; end plate offset, −500 V; Skimmer 1, 56.0 V; Skimmer 2, 6.0 V; capillary exit offset, 84.6 V; capillary exit, 140.6 V. Collision-induced dissociation (CID) spectra were obtained with a fragmentation amplitude of 1.00 V (MS/MS) using helium as the collision gas.

## 4. Conclusions

The present study was undertaken with one sample of each *Haplopappus* species used as “baylahuen”. For a quality control of the crude drugs and for metabolomic studies, much more samples should be included to represent the qualitative and quantitative variations within the same species. Additional studies should be carried out, including quantification of the single constituents and molecular biology for a better differentiation of the species and possible hybrids. This report compares the phenolic composition of the main sources of baylahuen tea, including both the infusion and MeOH extract, and provides, for the first time, an insight into the chemical diversity of the herbal tea. This information can be useful to design appropriate management strategies of the plant, as massive collection for commercial purposes has led to significant decreases in the wild growing population of this appreciated medicinal plant.

The method presented allowed a clear differentiation of the different *Haplopappus* species commercialized as the sources of “baylahuen” tea and a rapid identification of the main constituents. The antioxidant effect of the extracts, as determined by the DPPH assay, indicate that *H. multifolius* and *H. taeda* are the best source of free radical scavengers. As the *w/w* extraction yields of *H. taeda* are higher than those of *H. multifolius*, *H. taeda* is a better source of antioxidants. This trend is in agreement with the total phenolic and flavonoid content of the samples. The results obtained suggest that the regular consumption of teas prepared from these herbs may be useful against certain pathologically-relevant oxidant species.
